# Around the Hospitals

**Published:** 1893-11-25

**Authors:** 


### AROUND THE HOSPITALS.

Notice to the Managers of Institutions.—Special space is now reserved for the insertion of notices of hospital meetings and festivals. The Editor requests that all notices may reach The Hospital Office, 428, Strand, at least one week before the date of each meeting.

**The Doncaster Infirmary.**—At the annual meeting of this institution, the Secretary, Mr. W. Clark, who has held office for ten years, was appointed a life-governor and vice-president. The number of patients relieved by the institution have been rather fewer during the past than in the previous year, but the average residence per patient has been longer. The income amounted to £2,030.

**The Derbyshire Hospital for Sick Children.**—It would almost appear that there is something especially favourable towards the growth of hospitals in Derby. Last week we gave the account of the successful career of the Royal Infirmary, and we have now to show an equally good record of the Children's Hospital. Unusual generosity has been shown towards the infirmary, and yet this in no way effects the goodwill evinced on behalf of the smaller hospital. During the past year not only have the management been enabled to clear the revenue account of debt, but they can show a substantial balance on the right side. The capital account still bears a small debt, but this is evidently on the way of being erased, as Lord Burton (elected president for the coming year) has promised a hundred guineas for this purpose, provided others will subscribe the remaining sum. At the annual meeting many promises towards this were received, and we doubt not that next year we shall be able to record that the debt is entirely wiped out. During the past year annual subscriptions have increased, and the working classes, through the Hospital Thursday Collection, show an increased interest in the institution. The in-patients numbered 220 in the wards, and 1,619 in the out-patients' department. The institution is to be congratulated on the fact that the isolation ward was not required at all during the past year.

